# C5L2 CRISPR KO enhances dental pulp stem cell-mediated dentinogenesis via TrkB under TNFα-induced inflammation

**DOI:** 10.3389/fcell.2024.1338419

**Published:** 2024-01-22

**Authors:** Muhammad Irfan, Hassan Marzban, Seung Chung

**Affiliations:** ^1^ Department of Oral Biology, College of Dentistry, University of Illinois Chicago, Chicago, IL, United States; ^2^ Department of Human Anatomy and Cell Science, University of Manitoba, Winnipeg, MB, Canada

**Keywords:** complement C5a, C5L2, dentinogenesis, DPSC, TrkB, inflammation

## Abstract

**Background and Objectives:** Dental caries is one of the most common human pathological conditions resulting from the invasion of bacteria into the dentin. Current treatment options are limited. In many cases, endodontic therapy leads to permanent pulp tissue loss. Dentin–pulp complex regeneration involves dental pulp stem cells (DPSCs) that differentiate into odontoblast-like cells under an inflammatory context. However, limited information is available on how DPSC differentiation processes are affected under inflammatory environments. We identified the crucial role of complement C5a and its receptor C5aR in the inflammation-induced odontoblastic DPSC differentiation.

**Methodology:** Here, we further investigated the role of a second and controversial C5a receptor, C5L2, in this process and explored the underlying mechanism. Human DPSCs were examined during 7-, 10-, and 14-day odontogenic differentiation treated with TNFα, C5L2 CRISPR, and tyrosine receptor kinase B (TrkB) antagonist [cyclotraxin-B (CTX-B)].

**Results:** Our data demonstrate that C5L2 CRISPR knockout (KO) enhances mineralization in TNFα-stimulated differentiating DPSCs. We further confirmed that C5L2 CRISPR KO significantly enhances dentin sialophosphoprotein (DSPP) and dentin matrix protein-1 (DMP-1) expression after 14-day odontoblastic DPSC differentiation, and treatment with CTX-B abolished the TNFα/C5L2 CRISPR KO-induced DSPP and DMP-1 increase, suggesting TrkB’s critical role in this process.

**Conclusion and Key applications:** Our data suggest a regulatory role of C5L2 and TrkB in the TNFα-induced odontogenic DPSC differentiation. This study may provide a useful tool to understand the mechanisms of the role of inflammation in dentinogenesis that is required for successful DPSC engineering strategies.

## Graphical Abstract

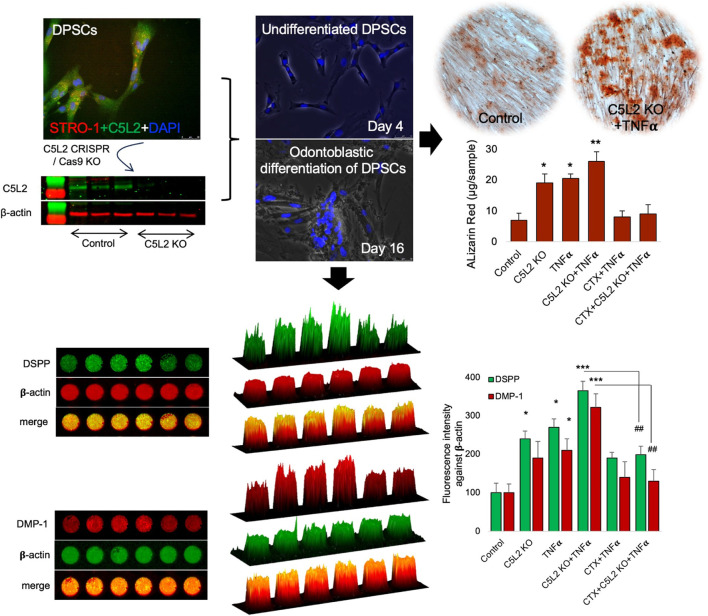

## 1 Introduction

Stem cell therapy and tissue engineering are considered among recent advances in regenerative medicine (Jiang et al., 2017). They have been used extensively in various pathological conditions for tissue regeneration and organ repair such as bone, ligament, and the heart (Mimeault and Batra, 2006; Caplan, 2007). Regenerative medicine focuses on the piecemeal reformation of *in vitro* procedures that nature uses during the morphogenesis of a given organoid, and here, stem cell research offers huge potential for maintaining homeostasis, regeneration, and repair (Abdel Meguid et al., 2018). Human DPSCs are derived from the neural crest and can be efficiently used for regeneration because of their easy accessibility with minimum invasion, lower immunogenicity, and, therefore, minimum rates of tissue rejection (Huang et al., 2009; Sakai et al., 2012). They are broadly recognized as stem cells with the most potential for tooth regeneration as they differentiate into osteoblasts, odontoblasts, and chondrocytes and also play an important role in pulp revascularization (Rombouts et al., 2017).

Caries is one of the major dental health issues affecting the majority of the US population (Islam et al., 2007). The dentin–pulp complex response is determined by the severity of the injury; e.g., moderate injury involves odontoblasts, which produce protective reactionary dentin (Chogle et al., 2012; Couve et al., 2014), while in case of serious injury, full or partial regeneration occurs, including vascularization, innervation, and dentin repair triggered by odontoblast-like cells (About, 2011). It may cause severe pain and require endodontic therapy or might lead to permanent tooth loss (Edwards and Kanjirath, 2010). Among several culprits behind the caries is the physiochemical dissolution of the teeth and bacteria, while bacteria or bacterial toxin interaction with DPSCs initiates the reparative process of tertiary dentin repair (Conrads, 2018).

The complement system, a key player in innate immunity, is expressed and activated in carious teeth injury or during inflammation, which is also known to assist the regeneration process (Chmilewsky et al., 2014a). Complement C5a receptor (C5aR1) has been known for its favorable effects on the rejuvenation of numerous tissues including the bone, heart, and liver (Mastellos et al., 2001; Ignatius et al., 2011; Lara-Astiaso et al., 2012). Recently, we have studied the role of C5aR1 in tooth regeneration (Pasiewicz et al., 2021; Irfan et al., 2022a; Kim et al., 2023a) and DPSC-mediated brain-derived neurotrophic factor (BDNF) modulation (Irfan et al., 2022b). Another C5a-like receptor 2 (C5aR2, also known as C5L2) has been cloned and is still considered as a controversial and dubious receptor; it is known to work against C5aR1, but there is limited available information. Previously, we explored C5L2’s effects on DPSCs in BDNF and dentin matrix protein-1 (DMP-1) modulation (8 h effect) using siRNA (Chmilewsky et al., 2019; Irfan and Chung, 2023). One of the limitations of siRNAs is their short-term effects, so they are not suitable for evaluating their effects on the long-term dentino-differentiation process, which usually takes about 14 days. Here, we seek to identify the comprehensive role of C5L2 on the odontoblastic differentiation of DPSCs and its mineralization using permanent C5L2 CRISPR/Cas9 KO.

## 2 Materials and methods

### 2.1 Chemicals and reagents

Human DPSCs were purchased from Lonza Pharma & Biotech (Cat. # PT-5025). MEM-alpha, DMEM, PBS, fetal bovine serum, L-glutamine, and antibiotic–antimycotic solution were procured from Gibco™ Fisher Scientific (Waltham, MA, United States). Poly-D-Lysine (Bio-Coat™, 12 mm)-coated round German glass coverslips were purchased from Corning™ Fisher Scientific (Cat. # 354087; Waltham, MA, United States). RIPA buffer was from Cell Signaling (Danvers, MA, United States), the DMP-1 ELISA kit was from Invitrogen, Thermo Fisher Scientific (Waltham, MA, United States), and the DSPP ELISA kit was from MyBioSource (San Diego, CA, United States). Various antibodies were procured: anti-C5L2 was from BioLegend (San Diego, CA, United States), anti-DMP-1, anti-DSPP, and anti-β-actin were from Fisher Scientific (Waltham, MA, United States), and anti-STRO-1 was from Santa Cruz (Dallas, Texas, United States). Fluorescent secondary antibodies were from Life Technologies (Grand Island, NY, United States). Human recombinant TNFα was from Invitrogen, Thermo Fisher Scientific (Waltham, MA, United States), and a few other chemicals were from Fisher Chemical (Nazareth, PA, United States). C5L2 CRISPR/Cas9 KO plasmid human (sc-410578) and reagent system were purchased from Santa Cruz Biotechnology (Dallas, TX, United States).

### 2.2 Cell culture

Commercially available human DPSCs, which were guaranteed through 10 population doublings, to express CD105, CD166, CD29, CD90, and CD73 and to not express CD34, CD45, and CD133, were further evaluated by immunocytochemistry in cultures with the STRO-1, a stem cell marker. DPSCs were cultured at 37°C and 5% CO2 in regular/osteogenic media for 72 h in regular growth media (α MEM containing 10% fetal bovine serum (FBS) and 1% L-glutamine), and then, cells were transfected with CRISPR/Cas9 C5L2 KO, and successful transfection was confirmed by Western blot. Then, cells were swapped with dentinogenic media (DMEM containing 20% FBS, 1% L-glutamine, and antimycotic/antibiotic, i.e., 100 μg/mL streptomycin and 100 U/mL penicillin, supplemented with 100 μg/mL ascorbic acid, 10 mmol/L β-glycerophosphate, and 10 mmol/L dexamethasone) for further 10-day differentiation and treated with TrkB inhibitor (cyclotraxin-B: CTX; 200 nM) or TNFα (10 ng/mL) every 3 days. All the experiments were conducted with different sets of DPSCs (between the second and fourth passages) three times, and cell proliferation was measured by counting the total number of cells.

### 2.3 C5L2 CRISPR/Cas9 KO

Human DPSCs were grown in a six-well plate culture chamber in 2 mL of free-antibiotic medium up to 60% confluence, and then, transient transfection with CRISPR/Cas9 KO was performed using the C5L2 CRISPR/Cas9 KO plasmid (h) (sc-410578), according to the manufacturer’s protocol. Cells were incubated at 37°C in a CO_2_ incubator in 2 mL of antibiotic-free and serum-free transfection solution containing a mixture transfection reagent and 0.75 μg/mL of C5L2 plasmid. After an incubation of 2 days, transfection was confirmed by Western blotting. Then, the medium was aspirated and replaced with fresh dentinogenic media every 3 days.

### 2.4 Real-time PCR

Human DPSCs were cultured in a six-well plate at 5 × 10^4^ cells/well, up to 7 days in dentinogenic media. The total mRNA was extracted using the RNeasy Mini Kit (QIAGEN, Hilden, Germany) and analyzed using the Fisher Scientific NanoDrop 2000 device. The cDNA samples were analyzed using the Applied Biosystems SYBR green reagent system, according to the manufacture’s protocol. Primer sequences were purchased from IDT. The expression levels of target genes were standardized by the housekeeping gene GAPDH using the 2^−ΔΔCt^ method (Livak and Schmittgen, 2001).

### 2.5 Immunocytochemistry

Human DPSCs were seeded at 1 × 10^4^ cells/well on 12-well glass culture chambers overnight before stimulation with TNFα. After required proliferation and differentiation (day 4 or day 16), cells were fixed with 4% paraformaldehyde and permeabilized using the Triton X-100 PBS solution. Then, cells were incubated overnight at 4°C with primary antibodies. The next day, cells were washed and incubated for 2 hours with a mixture of Alexa Fluor-594 anti-mouse IgG, Alexa Fluor-488 anti-rabbit IgG (2 μg/mL), and/or DAPI (1 μg/mL). The coverslips were sealed, and photographs were taken using a Leica DMI6000 B microscope. Fluorescence staining was statistically analyzed by determining the integrated density of each condition using ImageJ 1.49v software. A co-localization analysis was performed using both Colocalization Finder and JACoP plugins on ImageJ software.

### 2.6 Alizarin red staining

The six-well plates were washed two times with distilled water and fixed with 4% PFA for 1 hour at RT. The cells were then washed with distilled water two times, and 1 mL of 40 mM of alizarin red stain (ARS) was added per well; the ARS was provided by ScienCell (#8678). After 1 hour of gentle shaking, the plate was washed with distilled water (pH 4) three times and dried. The cells were inspected using a Leica DMi1 phase microscope, and images were obtained. Then, 10% acetic acid solution was added to the wells for 30 min at RT with gentle shaking, and samples were collected and processed with 10% ammonium hydroxide for ARS quantification. The plot was drawn against standards and respective samples.

### 2.7 Western blotting analysis

To see the effects of dentin sialophosphoprotein (DSPP) and DMP-1, cell lysates were prepared using RIPA buffer (50 mM Tris pH 7.6, 150 mM NaCl, 1% Triton X-100, 1 mM EDTA, 0.5% sodium deoxycholate, and 0.1% SDS) containing a protease inhibitor (Roche, Indianapolis, IN), and the protein was measured using the BCA method (Pierce™ BCA Protein Assay Kit, Thermo Fisher Scientific, Lenexa, KS). Equal amounts of protein (35 μg) were loaded and separated by SDS-polyacrylamide gel electrophoresis and transferred onto nitrocellulose (Bio-Rad, CA). Blots were probed using polyclonal antibodies specific for DSPP, DMP-1, or β-actin overnight at 4°C and then washed and probed with secondary antibodies for 2 h at RT. The protein bands were visualized by Odyssey CLx.

### 2.8 In-cell Western assay

Human DPSCs were seeded in growth media at 15 × 10^3^ cells/cm^2^ in 96-well plates. At sub-confluency, cells were incubated in an antibiotic-free medium and treated as mentioned above to KO and then differentiated in dentinogenic media. Then, cells were immediately fixed with 100% cold methanol (15 min) and saturated with 5% BSA (1.5 h). Cells were incubated overnight at 4°C with anti-DMP-1, anti-DSPP, or anti-β-actin. Cells were then washed (0.05% Tween-20/PBS) and incubated with respective IRDye-680RD or IRDye-800RD secondary antibodies (1 h) at RT. After five washes, plates were dried and scanned at 700 and/or 800 nm (Odyssey CLx).

### 2.9 DMP-1 and DSPP quantitative ELISA

Supernatants from DPSC culture, incubated with various abovementioned treatments, were collected from cultures after 7, 10, or 14 days of differentiation and assayed using the ELISA kit according to the manufacturer’s protocol. Then, a standard curve was constructed using standards and test samples in duplicate at increasing concentrations, and values were normalized accordingly.

### 2.10 Statistical analysis

The statistical analyses were performed on at least three independent experiments with duplicates or triplicates, and statistical significance was determined using one-way analysis of variance (ANOVA) followed by *post hoc* Dunnett’s test (SAS 9.4) to compare different treatments and their respective controls (*p*-value of 0.05 or less was considered statistically significant). In addition, the data were also analyzed by Tukey’s test for statistical significance between the groups. For the quantification of immunofluorescence staining intensity, ImageJ 1.49v software was used. Fixed areas of 1 mm × 1 mm or 2 mm × 2 mm were selected to analyze the number or fluorescence intensity of differentiated cells.

## 3 Results

### 3.1 DPSC culture and CRISPR/Cas9 C5L2 KO confirmation

**FIGURE 1:**
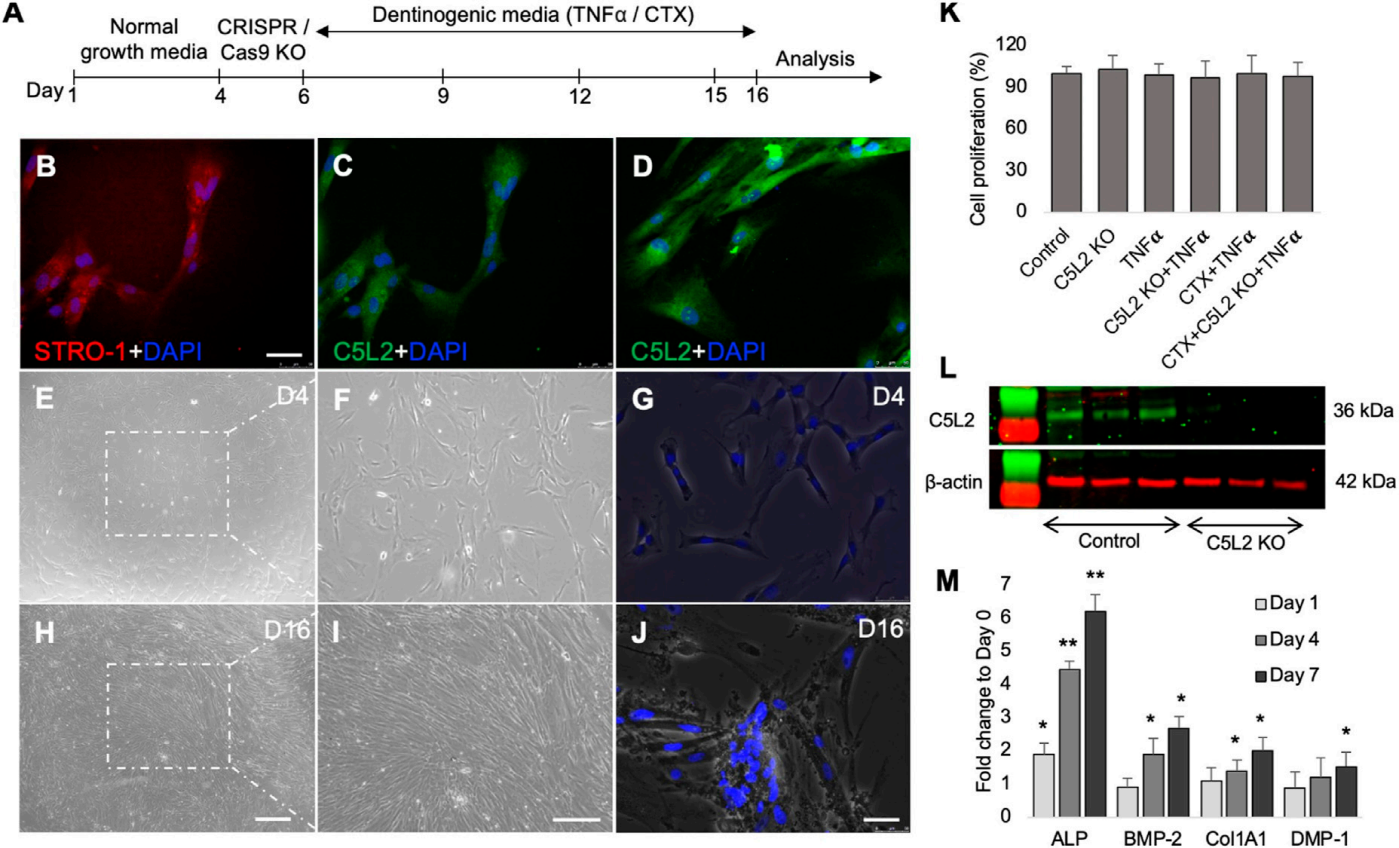
Differentiating DPSCs express C5L2, and TNFα stimulation enhances its expression. **(A)** Experimental scheme showing various stages of differentiation and treatments. **(B)** Commercial DPSCs were further confirmed with a mesenchymal stem cell marker STRO-1. Nuclei were counter-stained with DAPI. **(C)** DPSCs constitutively express C5L2. **(D)** TNFα stimulation enhances the expression of C5L2 [scale bar **(B–D)**: 50 μm]. **(E–J)** Cultured DPSCs showing various stages of differentiation in dentinogenic media under a light microscope (scale bar: 200 μm) or phase contrast microscopy [scale bar **(G, J)**: 50 μm]. **(K)** To evaluate whether various treatments affect the proliferation and differentiation rate, cell density was measured by cell counting, and no significant difference was found. The graph shows the mean ± SD of at least three independent experiments (*n* = 3) in duplicate. **(L)** To validate C5L2 CRISPR/Cas9 KO, a Western blotting assay was performed. C5L2 KO plasmid treated well clearly indicated C5L2 KO compared with control cells. **(M)** The expression of odontoblastic markers ALP, BMP-2, COL1A1, and DMP-1 mRNA during the odontogenic differentiation was quantified by real-time PCR. The elevated level of these markers represents the odontoblast-like differentiation of DPSCs. *p < 0.05 and **p < 0.01 vs. day 0-fold change. The bar graph shows the mean ± SD of at least three independent experiments (*n* = 3) in duplicate.

Our previous studies have shown that complement C5a and its receptor C5aR had a positive regulatory role in inflammation-induced odontoblastic differentiation of DPSCs (Irfan et al., 2022a; Pasiewicz et al., 2022). To identify the role of C5a’s second and controversial receptor, C5L2 in this process, DPSCs were cultured and treated according to scheme given in Figure 1A, and DPSCs were confirmed by the mesenchymal stem cell marker, i.e., STRO-1 (Figure 1B). These cells have been extensively used and validated in our previous publications (Irfan et al., 2022a; Irfan et al., 2022b; Kim et al., 2023b). It is already known that DPSCs subconstitutively express the C5L2 receptor, as shown in Figure 1C, while stimulation with TNFα enhances its expression (Figure 1D) as previously described by Chmilewsky et al. (2019). The inflammatory cytokine TNFα was used to mimic inflammatory environments (Tiegs and Horst, 2022). Differentiating DPSCs in dentinogenic media are shown at D4 (Figures 1E–G) and at D16 (Figures 1H–J) using a light microscope and fluorescent phase contrast microscope. Cells were transfected with C5L2 KO plasmids and treated with TNFα or TrkB antagonists (cyclotraxin-B; CTX). To determine whether these applications have effects on their proliferation or differentiation, cells were counted, and no significant difference was found among different treatment groups (Figure 1K). A successful C5L2 CRISPR/Cas9 KO was confirmed by Western blot, and bands clearly show the inhibition of C5L2 in DPSCs for further experimentation (Figure 1L). To validate the proposed *in vitro* model for studying odontoblast-like cell differentiation from DPSCs, alkaline phosphatase (ALP), bone morphogenic proteins 2 (BMP-2), type 1 collagen (Col1A1), and DMP-1 were analyzed using real-time PCR as previously described by Teti et al. (2013), and our results showed significant increment in their expression indicating the odontoblastic differentiation of DPSCs (Figure 1M; *p* < 0.05 and *p* < 0.01).

### 3.2 C5L2 KO enhances mineralization in TNFα-stimulated differentiating DPSCs

**FIGURE 2:**
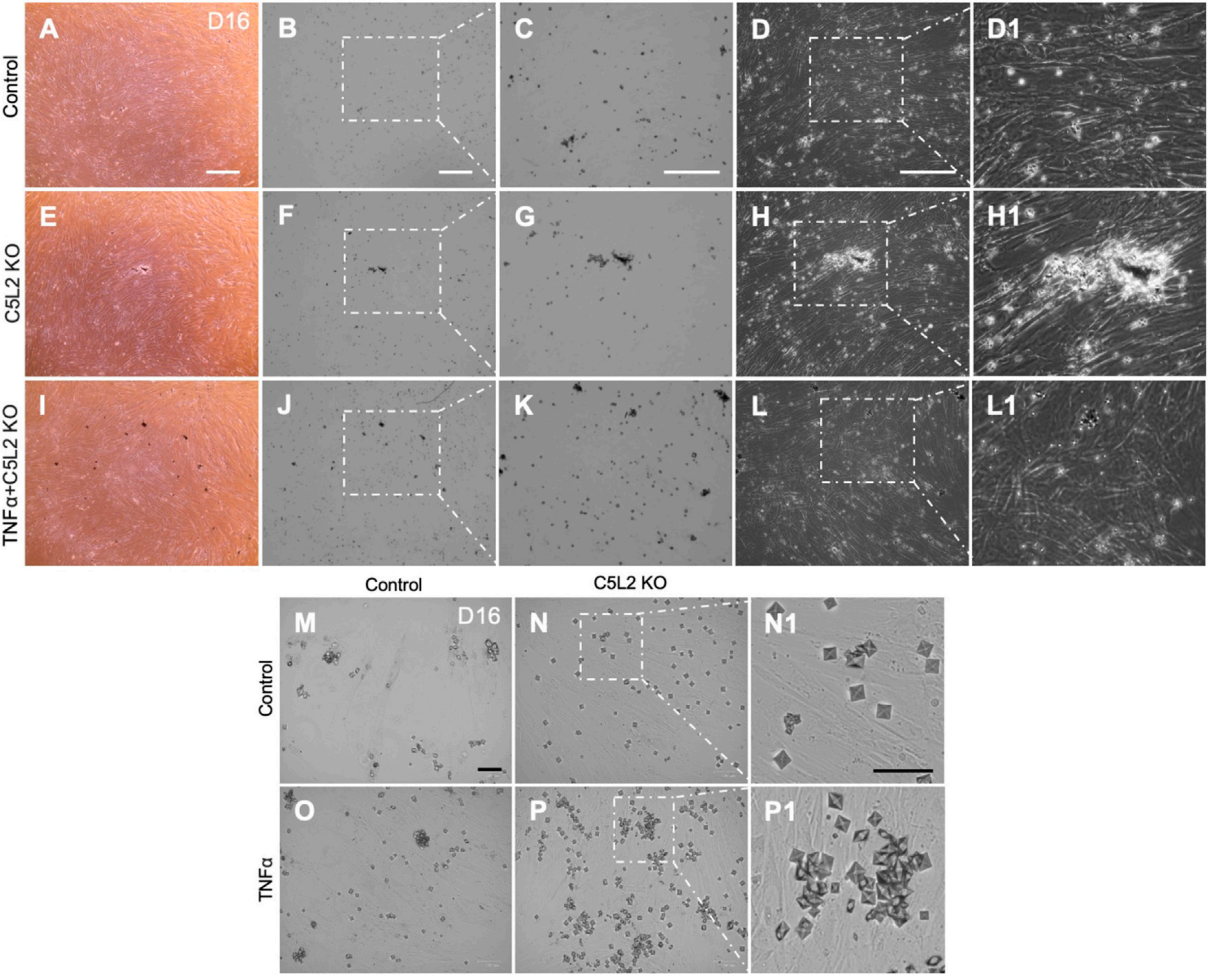
C5L2 KO DPSCs secrete more mineralized substances during odontoblastic differentiation. Control cells **(A–D)** show less mineralized substances than the C5L2 KO group **(E–H)** after 10 days of differentiation in dentinogenic media, while TNFα stimulation further enhanced the mineralization matrix **(I–L)**. **(M–P)** Images show calcium crystals in various treatment groups of differentiating DPSCs. TNFα-treated odontoblastic differentiating C5L2 KO DPSCs secrete more calcium crystals than C5L2 KO or TNFα alone compared with the control. The results shown are indicative of at least three independent experiments (*n* = 3) in duplicate.

DPSCs were subjected to differentiate in the dentinogenic medium for 10 days after C5L2 KO with TNFα stimulation. Under a light microscope observation, differentiated cells showed mineralization, analyzed by experts blinded to the study, which was further confirmed through various other following experiments. C5L2 KO groups showed more mineralization (Figures 2E–H) compared to the control differentiated group (Figures 2A–D). During stimulation with TNFα, differentiating DPSCs secreted more minerals (Figures 2I–L) than the control and C5L2 KO alone groups. It was further observed in higher magnification that calcium crystals are formed and abundantly found in the C5L2 KO group (Figures 2N, N1) and TNFα alone-treated group (Figure 2O) compared with the control group (Figure 2M), while crystallization significantly increased in the TNFα-stimulated C5L2 KO group (Figures 2P, P1).

### 3.3 ARS demonstrates an enhanced mineralization matrix in C5L2 KO cells

**FIGURE 3:**
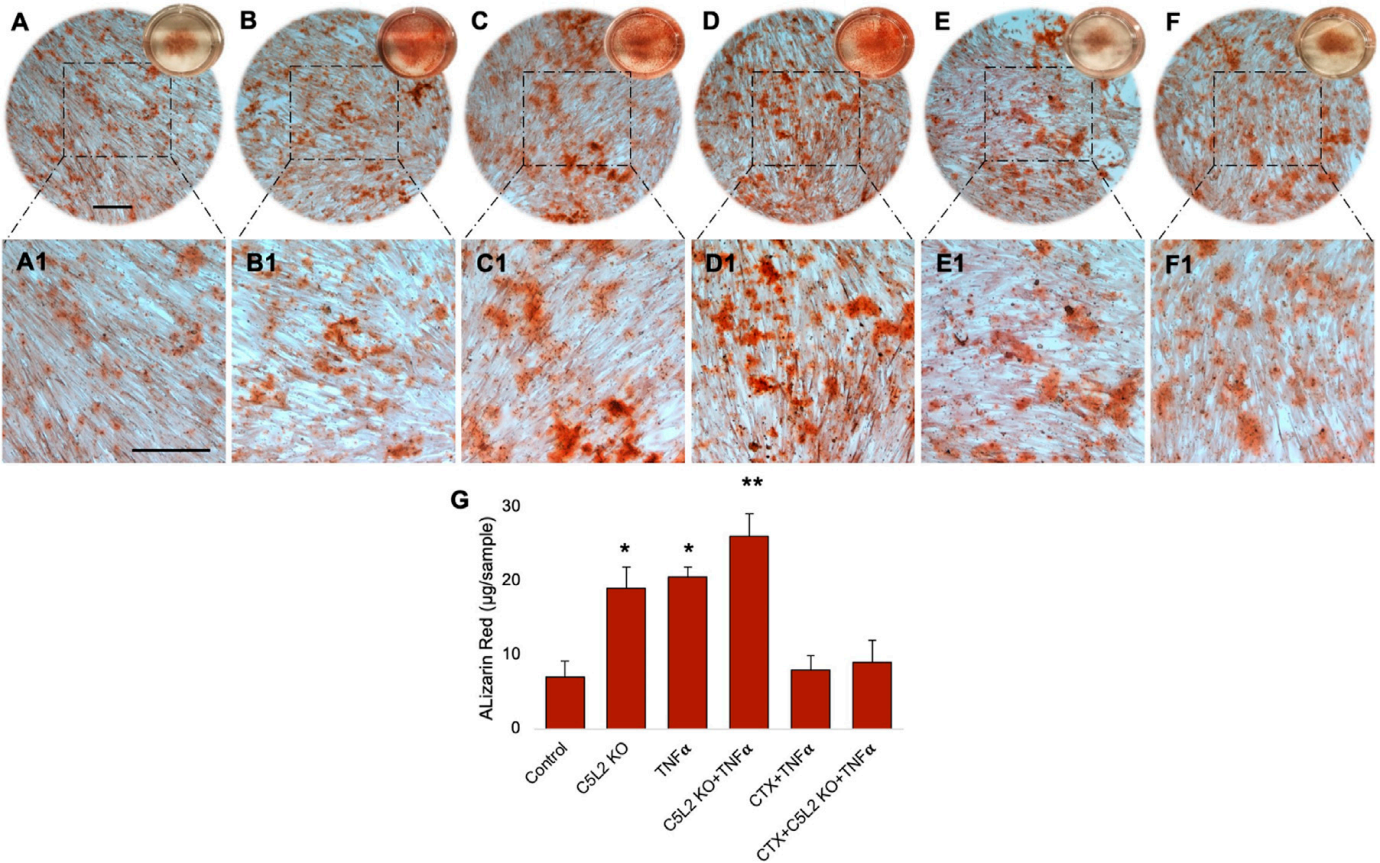
Mineralization activity of differentiated C5L2 KO DPSCs in dentinogenic media. **(A–F1)** Differentiated cells were stained with ARS, and images were taken using a Leica DMi1 phase microscope. Images show higher mineralization matrix in the C5L2 KO **(B, B1)** or TNFα alone **(C, C1)** or their combined treated group **(D, D1)** compared with the control **(A, A1)**, while the effects were reversed in the CTX-treated groups **(E–F1)**. Scale bars: 100 μm. **(G)** Bar graph shows ARS quantification among various treatment groups indicating significantly higher peaks in the C5L2 KO, TNFα, or their combined treatment, while CTX treatment reversed their effects. The graph shows the mean ± SD of at least three independent experiments (*n* = 3) in duplicate. *p < 0.05 and **p < 0.01 vs. control.

Cultured wells with differentiated DPSCs were stained with ARS to analyze the formation of a mineralization matrix surrounding the microenvironment during dentinogenic differentiation. The C5L2 KO group (Figures 3B, B1; *p* < 0.05) and TNFα group (Figures 3C, C1; *p* < 0.05) show more mineralization matrix than the control (Figures 3A, A1), while their combined effect drastically enhanced mineralization phenotypes (Figures 3D, D1; *p* < 0.01).

Our studies demonstrated that BDNF-TrkB receptor signaling affects DPSC odontoblastic differentiation under an inflammatory context (Kim et al., 2023b). In addition, C5L2 directly regulates BDNF modulations on DPSCs (Irfan and Chung, 2023) suggesting the possible link between them. To demonstrate the specific role of TrkB in DPSC differentiation under TNFα/C5L2 KO-treated conditions, cells were treated with a TrkB inhibitor CTX. Interestingly, CTX treatment reversed the effects of TNFα and C5L2 KO (Figures 3E–F1) indicating the involvement of TrkB in TNFα/C5L2 KO-mediated mineralization (Bernar et al., 2022).

Our results clearly show significant differences among the various treatment groups (Figure 3G) indicating that TNFα and C5L2 KO have potentially triggered the mineralization matrix in the surrounding microenvironment.

### 3.4 TNFα/C5L2 KO significantly enhances DSPP and DMP-1 secretion from differentiating DPSCs, and it is dependent on TrkB

**FIGURE 4:**
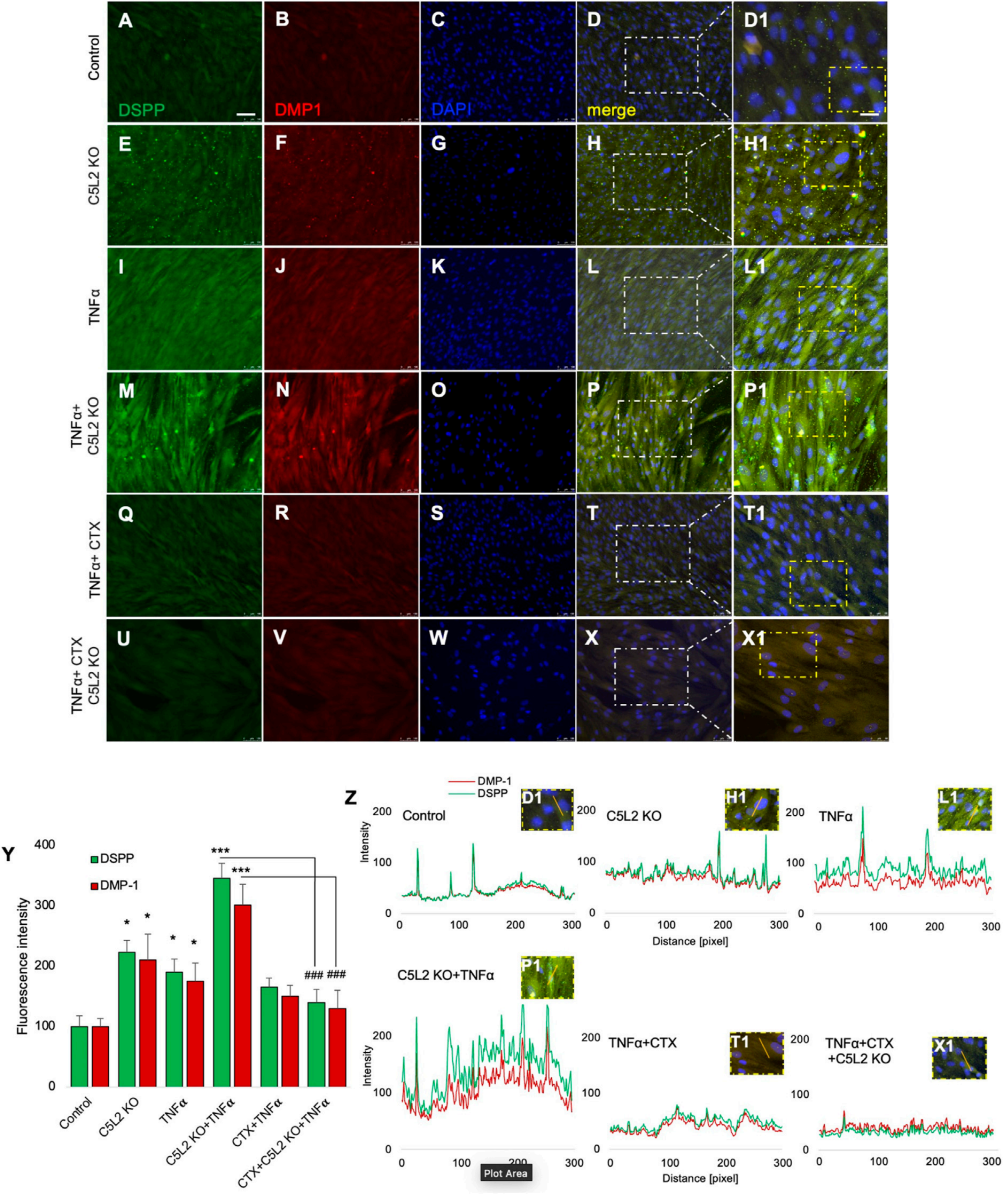
Effects of C5L2 KO on mineralization markers DSPP and DMP-1. **(A–X)** Double fluorescent immunostaining was performed (DSPP: green; DMP-1: red) after 10 days of DPSC odontoblastic differentiation in dentinogenic media with or without TNFα or CTX in C5L2 KO cells. Cells were counter-stained with DAPI (blue). **(A–D)** Control cells showed less DSPP and DMP-1 expression than the C5L2 KO **(E–H)** or TNFα alone **(I–L)**-treated groups, while their combined effect is significantly higher **(M–P)**. CTX treatment significantly abolished the effect of C5L2 KO or TNFα **(Q–X)**. **(Y)** The bar graph shows a significant increment in DSPP and DMP-1 expression, and even more enhanced expression in the combined treated group was reversed by CTX treatment. The graph shows the mean ± SD of at least three independent experiments (*n* = 3) in duplicate. *p < 0.05 and ***p < 0.001 vs. control. ^###^ *p* < 0.001 vs. C5L2 KO and TNFα-combined group **(Z)**. Line graph showing the co-localization of DSPP and DMP-1 being the highest peaks observed in C5L2 KO and TNFα-treated groups.

To further confirm the role of C5L2 in DPSC dentinogenic differentiation, DPSCs were cultured and differentiated for 10 days in dentinogenic media after C5L2 KO. Cells were treated with TNFα or CTX every 3 days as mentioned above. It is known that differentiated cells show odontoblastic-like properties as they express DSPP and DMP-1, which are well-established odontogenic markers (Demarco et al., 2010; Sabbagh et al., 2020). Our results show that the C5L2 KO group (Figures 4E–H1) and TNFα-treated cell group (Figures 4I–L1) show higher expression of DSPP and DMP-1 compared with the control cells (Figures 4A–D1), while their combined effect even more enhanced the expression of mineralization markers (Figures 4M–P1). Consistent with the mineralization assay (Figures 3, 4), CTX treatment significantly abolished the effects of TNFα (Figure 4Q-T1) and C5L2 KO (Figures 4U–X1). The bar graph (Figure 4Y) shows a significant increment in DSPP and DMP-1 expression in TNFα and C5L2 alone (*p* < 0.05) and their co-treatment (*p* < 0.001), while CTX treatment reversed the effect of TNFα or C5L2 KO, suggesting the role of TrkB in the modulation of these mineralization marker proteins.

Figure 4Z shows the line graph for the co-localization of DSPP and DMP-1. Figures were analyzed using ZEN (blue) software, and graphs were created. The graphs clearly show the significantly enhanced expression of DSPP and DMP-1 in the co-treated group of TNFα and C5L2 KO compared with other groups, indicating that TNFα and C5L2 KO modulate the expression of odontoblastic mineralization markers.

**FIGURE 5:**
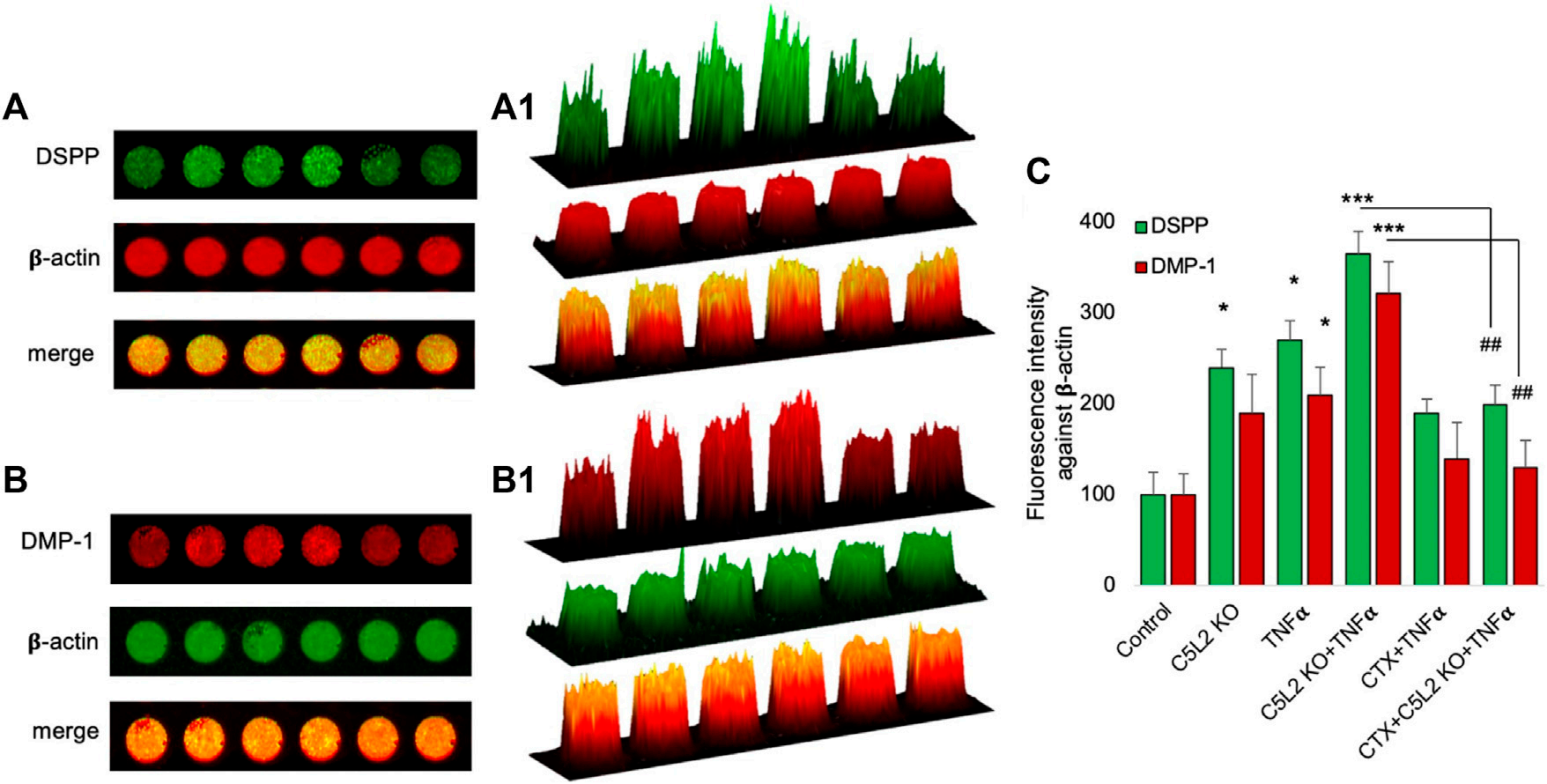
In-cell Western assay showing the expression of DSPP and DMP-1 in various treatment groups with or without TNFα or CTX in the C5L2 KO or control odontoblastic differentiated DPSCs. **(A, B)** Cells were assayed by the in-cell Western technique and photographed using Odyssey CLx; C5L2 KO and TNFα showed higher intensities under red and green channels against beta-actin. **(A1–B1)** 2.5D model shows the highest peaks among the C5L2 KO and TNFα alone or combined treated group. **(C)** The bar graph shows the increased expression of DSPP and DMP-1 in C5L2 KO and TNFα-treated groups. The graph shows the mean ± SD of at least three independent experiments (*n* = 3) in duplicate. *p < 0.05 and ***p < 0.001 vs. control. ^##^ *p* < 0.01 vs. C5L2 KO and TNFα-combined groups.

Similarly, the in-cell Western assay showed an increment in the C5L2 KO and TNFα-treated groups, while there was significant secretion of DSPP and DMP-1 in the co-treated group compared to the control (Figures 5A, B). Figures 5A1, B1 show 2.5D model graphs depicting high peaks in the TNFα and C5L2 KO groups and lower peaks in the CTX-treated groups, indicating the abolishing effect of the TrkB antagonist on DSPP and DMP-1. Similarly, the bar graph shows a significant difference between the TNFα and C5L2 KO groups (*p* < 0.05) and co-treated groups (*p* < 0.001) against the control, while CTX reversed the effects of TNFα and C5L2 KO (Figure 5C).

### 3.5 DSPP and DMP-1 quantification confirms the positive role of C5L2 KO on dentinogenic markers

**FIGURE 6:**
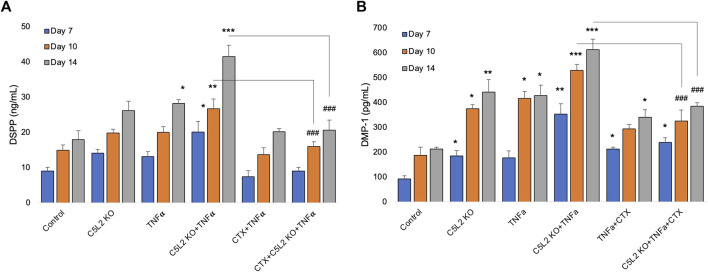
Effects of C5L2 KO or TNFα on the secretion of DSPP and DMP-1 in supernatants of differentiated DPSCs. **(A, B)** Cells were cultured and incubated with various treatments such as C5L2 KO plasmid, TNFα alone, or combined with CTX. The supernatants (conditioned media) were collected at days 7, 10, and 14 during differentiation, and an ELISA was performed to quantify DSPP and DMP-1 secretion in C5L2-mediated DPSCs according to the manufacturer’s protocol. **(A)** In the supernatant, DSPP and DMP-1 production was significantly increased in the C5L2 KO cells with or without TNFα at all stages. However, CTX treatment reversed the DSPP and DMP-1 production. The graph shows the mean ± SD of at least three independent experiments (*n* = 3) in duplicate. *p < 0.05, **p < 0.01, and ***p < 0.001 vs. control; ###*p* < 0.001 vs. the respective line-indicated group.

To complement immunocytochemistry and in-cell Western data, we performed ELISA. Our data show C5L2-mediated DSPP and DMP-1 regulations in odontoblastic differentiating DPSCs at different stages (Figure 6). Supernatants were collected at D7, D10, and D14 and analyzed. DSPP and DMP-1 productions were observed increasingly at various stages from day 7 to day 14. The co-treated group showed the most significant production of DSPP and DMP-1 in the TNFα and C5L2 KO (*p* < 0.001) groups compared with alone (*p* < 0.05) or control groups, while the CTX treatment significantly abolished the effect of TNFα or C5L2 KO (*p* < 0.001). These results suggest that C5L2 KO odontoblastic differentiating DPSCs secrete higher levels of mineralization markers via modulating TrkB receptors, and TNFα stimulation enhances the effect of C5L2 KO.

## 4 Discussion

In this study, we first successfully generated permanent C5L2 CRISPR/Cas9 KO DPSCs to overcome the limitations of C5L2 siRNA. Our results demonstrate that C5L2 constitutes a negative regulator of odontogenic DPSC differentiation as the C5L2 knockout induces a significant upregulation of mineralization and odontogenic lineage gene expression such as DMP-1 and DSPP. Consistent with our previous studies (Kim et al., 2023a; Pasiewicz et al., 2022), inflammation enhanced C5L2 KO-mediated DPSC odontoblastic differentiation. We further identified an underlying mechanism that treatment with TrkB antagonist CTX-B abolished the TNFα/C5L2 KO-induced DSPP and DMP-1 expression indicating TrkB’s critical role in this process.

Stem cell therapy is a potent and promising strategy in regenerative dentistry aiming to regenerate tertiary dentin or the dentin–pulp complex, allowing the conservation and restoration of functional teeth (Nakashima and Iohara, 2014). DPSCs play critical roles in dental–pulp complex regeneration. Along with their main roles in dentin formation, for example, they are also involved in critical steps of revascularization in the damaged pulp. The vascular system helps waste removal and nutrient supply during pulp inflammatory response and consequent regeneration. Hypoxic insult leads to the secretion of vascular endothelial growth factor (VEGF) from DPSCs which has been applied in tissue engineering (Rombouts et al., 2017; Mattei et al., 2021; Delle Monache et al., 2022).

It is generally accepted that there is a strong linkage between dental–pulp complex regeneration and inflammation, especially given that dental caries/regeneration occurs in an inflammatory context. Inflammation and complement system activation are natural and innate host immune responses to injury, which, in the case of caries, appeal to pulp progenitor cell migration, proliferation, and odontoblastic differentiation (Chmilewsky et al., 2013; Chmilewsky et al., 2014a; Chmilewsky et al., 2014b). This is considered one of the most efficient primary reactions against pathogens or injury, including recruiting the immune cells due to the secretion of critical anaphylatoxins C5a and C5b (Cruvinel et al., 2010). However, hyperactive inflammation due to bacteria or toxins could damage the tissue, e.g., during advanced caries affecting dentin (Shivakumar et al., 2009); therefore, new odontoblasts or like cells can assist in dentin repair and regeneration (Goldberg et al., 2008), and this complex regenerative course is primarily allied with DPSCs and their key factors involved in differentiation (Chmilewsky et al., 2014a). Previously, we have demonstrated the roles of C5aR-mediated DPSCs in neural regeneration and dentinogenesis (Irfan et al., 2022a; Irfan et al., 2022b; Pasiewicz et al., 2022; Kim et al., 2023a), while little is known about the role of C5L2 due to its controversiality (Li et al., 2013; Chmilewsky et al., 2019; Irfan and Chung, 2023). We previously used C5L2 siRNA to evaluate its role in DMP-1 modulation and demonstrated the negative role of C5L2 in DMP-1 expression (Chmilewsky et al., 2019). Since siRNAs have restricted effects with a short-affected time window, here, we ought to permanently knock out C5L2 by CRISPR/Cas9 KO plasmid and explore its detailed effects on sustained odontoblastic differentiation and mineralization matrix. Our results showed that C5L2 KO DPSCs show more mineralization matrix than control cells, and its mineralization was further enhanced when cells were stimulated with TNFα. TNFα-mediated enhancement is consistent with our previous studies showing that inflammatory stimulation such as LTA and LPS facilitates odontoblastic pulp fibroblasts or DPSC differentiation via p38 and C5aR or C5L2 (Chmilewsky et al., 2017; Irfan et al., 2022a; Kim et al., 2023a; Kim et al., 2023b), implying the role of inflammation in dentinogenesis. Similarly, Simon et al. (2010) and Qin et al. (2012) also documented the role of p38^MAPK^ in odontoblastic differentiation and dentinogenesis.

A previous study has shown that TNFα-stimulated DPSCs constitutively express C5L2 (Chmilewsky et al., 2019); we stimulated C5L2 KO DPSCs with TNFα and found that it enhanced the expression of mineralization markers compared to those treated alone with TNFα or C5L2 KO cells, significantly higher than the control. Similarly, CTX treatment hampered the effects of TNFα alone or C5L2 KO cells combined with TNFα and reversed their effects drastically. The effects have been observed through various experimental techniques, and a trend was drawn similar to each of the parameters such as fluorescent expression, ARS, in-cell Western assay, and quantitative ELISA, suggesting the role of C5L2 and TrkB in TNFα-stimulated DPSC odontoblastic differentiation. Our study used well-known dentinogenesis markers—DSPP and DMP-1—to confirm our conclusion. DSPP plays a critical role in cell signaling and is known as an inducer of mineralization in the extracellular matrix (Sfeir et al., 2011). It is also known that DMP-1 is expressed (i) during the early to late stages of odontogenesis via the COL1A1 promoter or (ii) during the late stage of odontogenesis via the DSPP promoter (Lu et al., 2007). They are essential for the proper development of hard tissues, e.g., bones and teeth, and are known as positive regulators of hard tissue mineralization (Suzuki et al., 2012). Our DSPP and DMP-1 analyses demonstrated that C5L2 KO DPSCs significantly increased dentinogenesis, and TNFα stimulation further enhanced it.

**FIGURE 7:**
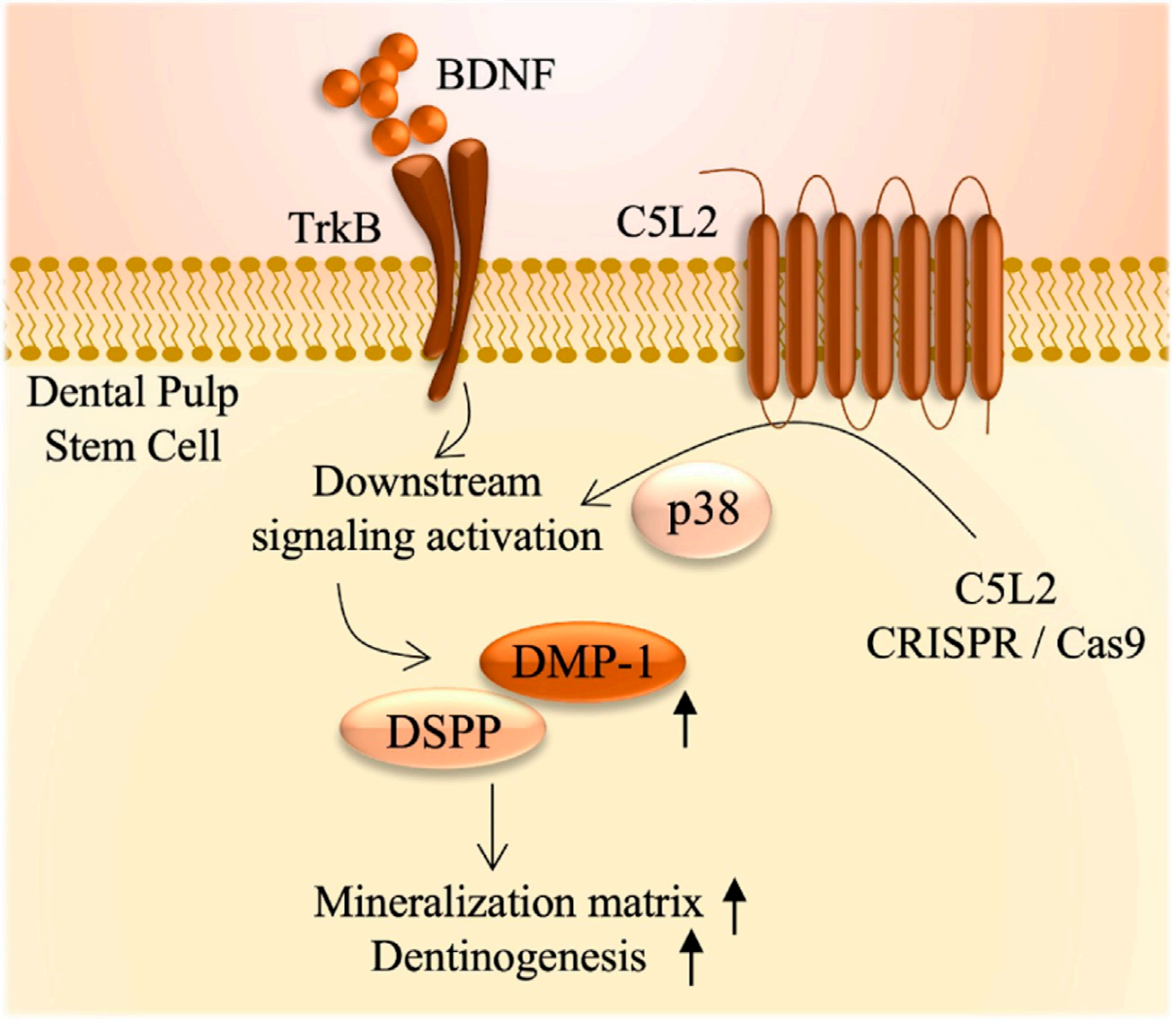
Summarized effects of C5L2 CRISPR/Cas9 KO on DPSCs.

We have recently shown that BDNF/TrkB is a crucial regulator in DSPP expression during odontoblastic DPSC differentiation (Kim et al., 2023b). BDNF is not only restricted to neurons but it has also been discovered in various other tissues, including cartilage, bone, tooth germ, and cardiac tissues (Bathina and Das, 2015). BDNF is a potent regulator of dentinogenesis; however, it has a very short half-life, i.e., 5–10 min, and scientists are finding alternative ways to get more of its beneficial effects in the various biological processes. Recently, we established that BDNF/TrkB is a critical regulator in the odontoblastic differentiation of DPSCs and expression of DSPP and DMP-1 (Kim et al., 2023b). Here, we evaluated the role of TrkB (a BDNF receptor) and explored the effect of the TrkB antagonist (CTX) on C5L2 KO DPSCs and found that it modulates the expression of DSPP and DMP-1, suggesting its involvement in the inflammatory odontoblastic differentiation of DPSCs and, consequently, adding up to the mineralization matrix (Figure 7). Alizarin red staining is considered as the gold standard to analyze the mineralization matrix (Bernar et al., 2022), and our results show that TNFα-stimulated C5L2 KO cells stained more with ARS, and ARS quantification further confirms the phenomenon.

In a nutshell, our data suggest the role of C5L2 and TrkB in the TNFα-stimulated DPSC odontoblastic differentiation by increasing the expression of DSPP and DMP-1, which contributes to the enhanced mineralization matrix.

## Data Availability

The raw data supporting the conclusion of this article will be made available by the authors, without undue reservation.
